# Persistent Cord Presentation Due to Marginal Cord Insertion at the Lower Edge of a Low-Lying Placenta: A Case of Successful Vaginal Delivery

**DOI:** 10.7759/cureus.84467

**Published:** 2025-05-20

**Authors:** Hitomi Kusakabe, Taito Miyamoto, Eriko Yasuda, Maya Komatsu, Masahito Takakura, Ayaka Yamaguchi, Yoshitsugu Chigusa, Masaki Mandai, Haruta Mogami

**Affiliations:** 1 Department of Gynecology and Obstetrics, Graduate School of Medicine, Kyoto University, Kyoto, JPN

**Keywords:** cord presentation, low-lying placenta, marginal cord insertion, preterm premature rupture of membranes, spontaneous vaginal delivery

## Abstract

The position of the umbilical cord within the uterus is influenced by its insertion site, with low insertion near the internal os raising concerns regarding the risk of cord prolapse and feasibility of vaginal delivery. This report describes a case of persistent cord presentation caused by marginal cord insertion at the lower edge of a low-lying placenta, further complicated by preterm premature rupture of membranes (pPROM) at 30 weeks. Given the fetal immaturity, expectant management was pursued despite the potential risk of cord prolapse. Although persistent amniotic fluid leakage occurred, no signs of fetal compromise or immediate cervical ripening were noted. Sequential transvaginal ultrasound examinations over a period of more than one month demonstrated a gradual resolution of cord presentation, likely facilitated by placental migration and descent of the fetal head shortly before the onset of labor. This progression ultimately enabled successful vaginal delivery at 35 weeks. The case reported herein highlights the critical role of close monitoring in managing persistent cord presentation associated with low cord insertion. Our findings suggest the possibility of avoiding cesarean delivery in similar high-risk scenarios.

## Introduction

The intrauterine position of the umbilical cord is influenced by various factors, including fetal presentation, location of the placenta, and site of cord insertion. In some cases, the cord is positioned below the presenting part of the fetus, a condition known as cord presentation; cord presentation poses a risk for cord prolapse, particularly in late pregnancy [1-5]. Although cord presentation associated with fetal malpresentation is relatively well-documented [6-8], cases in which the cord insertion point is near the internal os, and therefore the cord is inevitably positioned low, are rare, with few case reports available [9-11]. Notably, none of these reports has described the spontaneous resolution of cord presentation, as all cases have resulted in cesarean delivery. We present herein a case of persistent cord presentation resulting from marginal cord insertion at the lower edge of a low-lying placenta. In this case, the cord presentation ultimately resolved, facilitating successful vaginal delivery. This case provides two new insights into persistent cord presentation associated with low-lying cord insertion: a detailed chronological assessment of cord position changes and the knowledge that potential exists for spontaneous resolution of cord presentation, enabling vaginal delivery.

## Case presentation

A 39-year-old woman, gravida 3 para 1 abortion 1, noticed watery discharge at 30 weeks and four days of gestation. Her obstetric history included a term vaginal delivery at 41 weeks and one early miscarriage. She had no other notable past medical history. She conceived via in vitro fertilization and embryo transfer and was receiving antenatal care at another hospital, where the low-lying placenta was noted without other complications.

**Figure 1:**
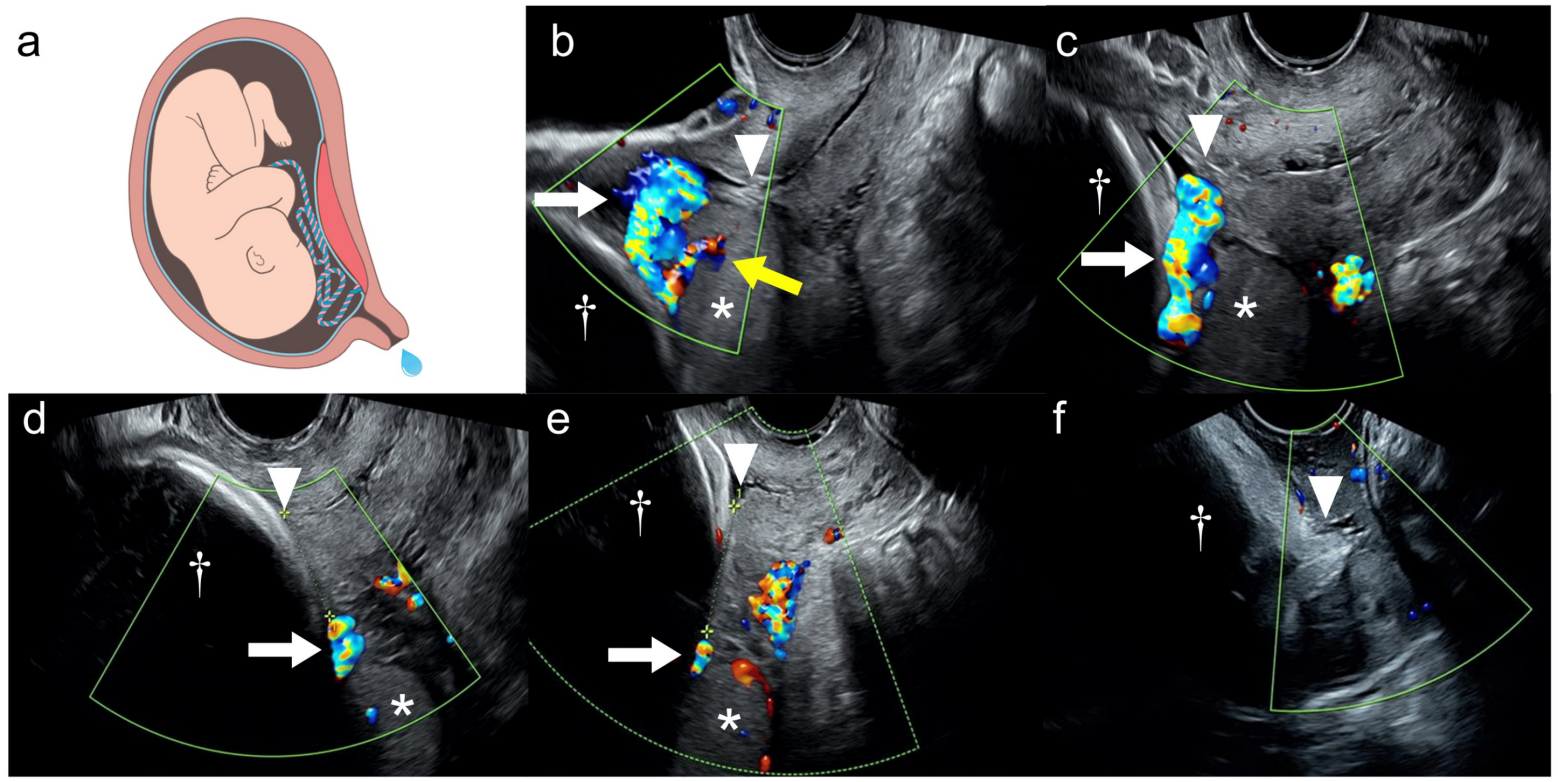
Schematic with ultrasound images captured at the time of emergency admission (a) Schematic of the fetus in utero, showing cord presentation caused by marginal cord insertion at the lower edge of a low-lying placenta complicated by premature rupture of membranes. This original illustration was created by SAIKOU, Inc., under commission from the authors. (b–f) Transvaginal color Doppler ultrasound images captured on (b) hospital day 1 (30 weeks and four days of gestation), (c) hospital day 8 (31 weeks and four days), (d) hospital day 30 (34 weeks and five days), (e) hospital day 32 (35 weeks and 0 days), and (f) hospital day 34 (35 weeks and two days, the day of delivery). In all images, * indicates the placenta, † indicates the fetal head, arrowheads mark the internal os, white arrows point to the umbilical cord, and the yellow arrow points to the insertion site of the cord into the placenta.

At emergency admission to our hospital, preterm premature rupture of membranes (pPROM) was confirmed by both visual inspection and positive nitrazine and insulin-like growth factor binding protein-1 tests. Transvaginal ultrasonography revealed that the fetus was in the cephalic presentation, the cervical length was 37.4 mm, and the low-lying placenta was posteriorly located. Marginal cord insertion close to the internal os was identified, with the cord positioned between the fetal head and the internal os (Figures 1a, 1b).

Vasa previa was not observed. The estimated fetal weight was 1,390 g (−0.88 standard deviation), and no abnormalities were noted. A nonstress test showed no uterine contractions or fetal decelerations. Blood tests revealed no elevation in white blood cell count or C-reactive protein levels, indicating the absence of an inflammatory response.

Given the risks of cervical maturation and progression to cord prolapse, cesarean delivery was prepared as a contingency. Intramuscular betamethasone (12 mg for two days), prophylactic antibiotic treatment (intravenous ampicillin at 4 g/day and oral azithromycin at 250 mg/day), and prophylactic administration of ritodrine hydrochloride (4 mg/hour) to prevent uterine contractions were initiated. Uterine contractions, genital bleeding, or cervical shortening were still not observed. Owing to the immaturity of the fetus, continuing the pregnancy was deemed preferable, and expectant management with frequent evaluations was chosen.

**Figure 2:**
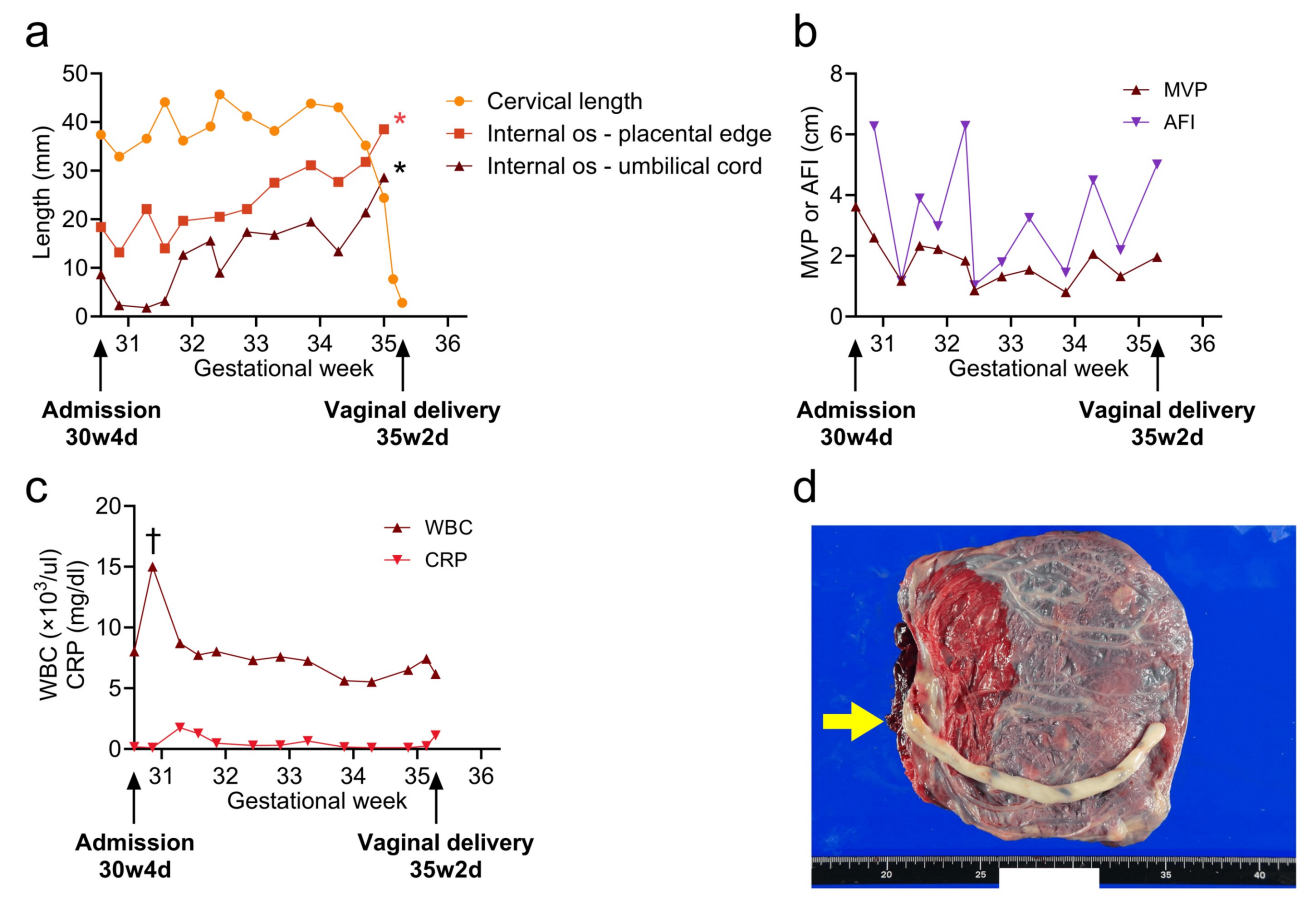
Chronological changes from admission to delivery and the placenta after delivery (a) Cervical length and distances from the internal cervical os to the edge of the placenta and the nearest edge of the umbilical cord; (b) Amniotic fluid volume, including the maximum vertical pocket (MVP) and amniotic fluid index (AFI); (c) White blood cell (WBC) counts and C-reactive protein (CRP) levels; (d) Macroscopic image of the placenta obtained after delivery; the yellow arrow indicates the insertion site of the cord into the placenta. The unit of the scale in the figure is centimeters. * The placenta and umbilical cord could not be visualized or measured using transvaginal ultrasonography beyond 35 weeks and one day; † The increase is considered to be due to betamethasone administration.

Over the subsequent weeks, the positions of the cord and placenta were monitored two to three times a week. Although cord presentation was still obvious at hospital day 8 (Figure 1c), the distance between the internal os and the placental edge or presenting cord increased over time (Figures 1d-1f; Figure 2a).

The volume of amniotic fluid fluctuated, but leakage never completely stopped (Figure 2b). No significant elevation in inflammatory markers was revealed through blood tests (Figure 2c), and administration of antibiotics was discontinued after seven days. Prophylactic tocolysis with ritodrine was ceased at 33 weeks and two days. Nonstress test results remained reassuring, and the fetal presentation remained cephalic throughout the course of observation.

At 34 weeks and five days, the patient developed light blood-streaked discharge of amniotic fluid and increasing uterine contractions. Ultrasonography revealed a cervical length of 35.2 mm, with the placenta and cord further from the internal os (31.8 mm and 21.4 mm, respectively; Figure 1d). At 35 weeks and 0 days, the cervical length shortened further to 24.4 mm, with the distances from the internal os to the placental edge and cord reaching 38.5 mm and 29.9 mm, respectively (Figure 1e). At 35 weeks and one day, cord presentation resolved entirely.

Spontaneous labor began at 35 weeks and two days. After confirming cephalic presentation and the absence of cord presentation (Figure 1f), vaginal delivery was achieved within one hour and 40 minutes; the cord did not prolapse. A female neonate weighing 2,272 g was delivered. The neonate’s Apgar scores were 8/9, and the pH of the umbilical artery was 7.329. The newborn required positive pressure ventilation for only a few minutes. An extended interval of two hours and 49 minutes between fetal and placental delivery was observed, which was associated with increased blood loss, totaling 3,156 g. The placenta was completely delivered with no retained tissue. Macroscopically marginal cord insertion was confirmed, which was consistent with ultrasonography findings (Figure 2d). The patient’s hemoglobin level was 7.2 g/dL on postpartum day 1, and no blood was transfused. The mother was discharged on postpartum day 5 and the infant on postpartum day 17, both without complications.

## Discussion

Two case series have summarized cord presentation observed in late pregnancy. Ezra et al. reported 13 cases identified during the third trimester [7], whereas Lange et al. documented nine cases occurring at or beyond 37 weeks of gestation [8]. Of these, 21 of the 22 cases were associated with fetal malpresentation at the time of detection; however, whether any of the cases involved low-lying placentas or low cord insertion is unclear. Including the present case, four cases of cord presentation associated with low cord insertion have been reported (Table 1) [9-11].

**Table 1:** Cases of cord presentation associated with low-lying cord insertion * Information not specified in the original paper; details were directly obtained from the author; † Cord insertion located at the lower edge of the low-lying placenta; ‡ Cord insertion site measured 5.6 cm from the internal os at 33 weeks. CS: cesarean section; N/A: not available; pPROM: preterm premature rupture of membranes; VD: vaginal delivery

Case	Maternal age at diagnosis (years)	Gestational age at diagnosis (weeks)	Low-lying placenta	Cord insertion	Fetal presentation at diagnosis	pPROM	Resolution of cord presentation before delivery	Gestational age at delivery (weeks)	Fetal presentation at delivery	Mode of delivery	CS indication
Uchide et al. (1997) [9]	32	27	Yes	Marginal^†^	Vertex	No	N/A	31	N/A	CS	Recurrent variable decelerations of the fetus
Oyelese et al. (2004) [10]	33	27	Yes	Marginal^†^	N/A	No	No	35	N/A	CS	Marked variable decelerations of the fetus
Mimura et al. (2020) [11]	43	14*	No	Velamentous^‡^	Vertex	No	No	38	Vertex	CS	Persistent cord presentation
Kusakabe et al. (2025) Present case	39	30	Yes	Marginal^†^	Vertex	Yes	Yes	35	Vertex	VD	N/A

Three of these cases involved marginal cord insertion at the lower edge of a low-lying placenta, and one involved velamentous cord insertion at a low insertion site. Our case was the only one complicated by pPROM. Among the three cases with follow-up ultrasonography, resolution of cord presentation was only observed in the present case. In three of the four cases, the mothers underwent cesarean section; the indications for the cesarean section were persistent cord presentation in one case and fetal deceleration in two cases. The cord did not prolapse in any of these cases.

Marginal and velamentous cord insertions located in the lower third of the uterus are associated with an increased incidence of variable decelerations and non-reassuring fetal status during labor, respectively, compared to insertions in the upper two-thirds [12]. Therefore, evaluating the cord insertion site is important, regardless of whether cord presentation is present. Indeed, two cases of low-lying placenta with marginal cord insertion have been reported, both of which exhibited fetal deceleration and required cesarean delivery [9,10]. In the current case, despite the coexistence of a low-lying placenta, marginal cord insertion, and pPROM, no signs of uterine contractions, infection, or complete loss of amniotic fluid were observed for a month. These factors likely prevented significant compression of the cord and deterioration in fetal status, thereby contributing to the avoidance of cesarean delivery for fetal indications.

In our case, persistent cord presentation was observed from 30 weeks of gestation, and the cord was no longer identified on transvaginal ultrasonography at 35 weeks and one day. As gestation progresses, low-lying placentas often migrate upward [13], possibly lifting the attached umbilical cord. Cervical ripening and subsequent descent of the fetal head can displace the presenting cord [14]. These mechanisms likely contributed to the resolution of cord presentation in our case. As evidenced by a case in which cord presentation persisted until 38 weeks [11], whether cord presentation will ultimately resolve with expectant management remains uncertain. Individualized treatment strategies based on continuous and frequent monitoring of the placenta and cord positions are essential.

## Conclusions

This case highlights the potential for spontaneous resolution of persistent cord presentation associated with marginal cord insertion at the lower edge of a low-lying placenta. Despite the presence of multiple high-risk factors, including cord presentation, low-lying placenta, and pPROM, careful expectant management with close monitoring enabled successful vaginal delivery without adverse maternal or neonatal outcomes. Serial ultrasonographic evaluation of cord and placental position was critical in guiding clinical decision-making. This case supports a flexible, case-by-case management strategy for cord presentation, where postponing cesarean delivery may be justified when fetal well-being is maintained and gestational age is not yet optimal.
